# Standards in Genomic Sciences: New beginnings to reflect the association between the journal and BMC

**DOI:** 10.1186/1944-3277-9-1

**Published:** 2014-12-08

**Authors:** George M Garrity

**Affiliations:** 1 Microbiology and Molecular Genetics, Department Michigan State University, 567 Wilson Rd, East Lansing MI 48824, USA

## 

Five years ago, we began the public phase of an experiment in open access publishing, aimed at addressing a perceived gap that was emerging in the field of genomics [1,2]. Companion papers, which were a staple of the field early on, were no longer considered an essential part because high-impact general interest journals no longer considered sequencing an entire genome a remarkable feat or worthy of publication. Even specialty journals would only publish such articles if they fit within their scope. The net result was that new genome sequences were rapidly accumulating in public databases but lacking formal publications that would inform readers of the rationale for why the sequence was generated, or a description of the methods used to produce and annotate the sequence and contextual information about the source organism, its habitat and its utility or its putative role in nature. While the potential value of viewing each genome as part of a larger collection rather than in isolation was obvious, the opportunity to do was quickly fading because there was no publication in which this information could be reported in a consistent and uniform manner [3]. In response to this need, the Genomic Standards Consortium (GSC) launched *Standards in Genomic Sciences* (SIGS) [4].

In the inaugural issue of SIGS, we introduced the concept of the Short Genome Report; a highly structured article that was designed to make comparison of genomes simple for both human and machine readers. We also introduced three other article types to fill other perceived needs: Standard Operating Procedures, Meeting Reports, and White Papers. Standard Operating Procedures (SOPs) were designed to provide detailed descriptions of sequencing and annotation pipelines so that readers could either duplicate results, or understand methodological differences that could have bearing on the outcome and interpretation of genome sequencing experiments. Meeting reports and white papers were introduced to fill a void in the scientific literature. This content is typically relegated to the "gray literature" by most publishers, and is neither indexed nor deposited in archives of the scientific literature. As a result, many important activities of communities of interest never become a part of the published record. The GSC sought to address this issue and to provide various communities of interest with a venue to report on their activities and concerns to funding agencies and to the broader scientific community in the pages of SIGS.

As we look back over the five years we have published SIGS, we are pleased to say that the experiment has been rather successful. The scientific community has responded positively and readership continues to expand (> 150 K readers from 193 countries/territories). This increase has been positively influenced by the inclusion of SIGS in the major indexes of scientific literature (PubMed Central, PubMed, Web of Science, Chemical Abstracts, Scopus, EBSCO). There have been over 275,000 downloads of articles from the SIGS home page and PubMed Central. Citation of SIGS articles in other publications also continues to increase steadily and now exceeds 3250. The impact factor (IF) of SIGS in 2014 was 3.17, and the five-year IF stands at 3.14. Contributions, which were initially from members and affiliates of the GSC have expanded well beyond that community, with submissions now coming from a number of groups in the US, Europe, Asia and Oceana.

Of course, much has changed in the field of genomics since SIGS began publishing. New sequencing and assembly methods have increased throughput and dramatically reduced the time and cost to generate genomic and metagenomic sequences, leading to new applications of sequence data to answer a variety of biological questions [5]. This has led to commoditization of these methods and increased the needs for standardization in data generation, analysis and interpretation.

A case in point has been the movement towards the use of genome sequence data in taxonomic circumscriptions of bacteria and archaea. One of the first such publications appeared in SIGS in 2010 [6] and has been followed by an additional 49 taxonomic proposals. This is a natural extension of the ongoing publication of type-strain genomes in SIGS arising from the *Genomic Encyclopedia of Bacteria and Archaea*[7,8]. To accommodate proposals for new species and new combinations of existing taxa, we are introducing an additional style of the Short Genome Report that includes the protologue and circumscription required in taxonomic proposals. This new style of genome report is specifically designed to ensure than any nomenclatural proposals will conform to the appropriate code of nomenclature. We are also introducing Extended Genome Reports to accommodate authors who wish to publish standardized genome comparisons or to provide more detailed information about a genome sequence(s) than was intended in a Short Genome Report. Other article types that will be introduced later this year include Short Single Cell Genome Reports and Standards for Reporting Metadata.

Changes in the field have also prompted us to revisit our publishing model. While the self-publishing model we initially adopted served as an excellent way to test our hypotheses, scaling SIGS to meet the needs of our authors and readers required a solution with a formal publishing infrastructure. We are delighted to partner with BioMed Central, who will be handling the administrative and production issues for this journal in the future. We believe this will also increase the visibility of articles published in SIGS.

As we bring the start-up phase of SIGS to a successful conclusion, we would like to express our sincere thanks to those organizations and individuals who helped to make the journal possible. These include Iain Gray, Vice President of Research and Graduate Studies of Michigan State University and the Michigan State University Foundation for providing the start-up funds, space and other resources that were essential in bringing SIGS to life; Oranmiyan Nelson, who served as the production editor of SIGS from 2009 to 2014; Charles Parker, who provided necessary operational support, including developing the XSL transformations used to produce the web version of our articles as well as modifications to the Open Journal Systems software we have used to publish the journal; the editors and reviewers for their service to the journal; the authors for their willingness to publish their work in a new Open Access publication; and the readers, who have willingly accepted SIGS as a part of the scholarly literature.
